# Inequalities in perceived health in the Russian Federation, 1994–2012

**DOI:** 10.1186/s12889-016-2810-x

**Published:** 2016-02-17

**Authors:** Pavitra Paul, Hannu Valtonen

**Affiliations:** Department of Health and Social Management, Faculty of Social Sciences and Business Studies, University of Eastern Finland, P.O. Box 1627, 70211 Kuopio, Finland

**Keywords:** Health inequalities, Concentration, Decomposition, Determinants, Self-perceived, Russia

## Abstract

**Background:**

Individual characteristics and socioeconomic strata (SES) are important determinants of health differences. We examine health inequalities in Russia and estimate the association of demography (gender and age) and SES (working status, income, geography of residence, living standard, wealth possession, and durable asset-holding) with perceived health over the period 1994–2012.

**Methods:**

This study uses nationally representative datasets from the Russian Longitudinal Monitoring Survey (RLMS: 1994–2012). We apply a random effect GLS model to examine the association of individual characteristics and individual heterogeneity in explaining self-perceived health status. In addition, we estimate a regression-based concentration index, which we decompose into the determinants of health inequalities.

**Results:**

The self-perceived health differences between the better-off and the worse-off is reduced over the 18 year period (1994 – 2012). The individual variances in self-perceived health status are higher compared to the variances between the individuals over the period. The measure of health inequality index (concentration index) indicates a change for better health for the better-off Russians. Being employed matters in perceiving a better health status for the Russians in 2012.

**Conclusions:**

Self-perceived health differences in the Russian Federation has changed over time. Such differences in changes are attributable to both changes in the distribution of the determinants of health as well as changes in the association between the determinants of health with the self-perceived health status. Though this study identifies the determinants of health inequalities for the Russians, the future research is to examine the in-country distribution of these determinants that produce health differences within the Russian Federation.

## Background

The economic development literature has highlighted the importance of health as a measure of societal well-being, especially in times of transition^1^ [1, 2]. In social epidemiology, the distribution of a population’s health is related to a causal triad: individual characteristics, geographical determinants, and changes over time. The WHO [3] model on the Social Determinants of Health posits socioeconomic position in population distribution of health.The association between socioeconomic strata (SES) and population health is already well-documented [4–8]. In development parlance, reduction of health inequalities is a public health policy mandate in both the national and the global context [9–13].

Standards of living and income distributions have taken centre stage in the discussion and measurement of the social determinants of population health [14–18]. Health inequality has been attributed to different dimensions of socioeconomic position, such as occupation, self-perceived societal position, education, and income or access to material resources [19]. Furthermore, in-country health difference are closely associated with the distribution of the population across SES – in other words, the in-country distribution of material deprivation reflects the in-country health differences, *ceteris paribus* [20–26]. The health outcomes and patterns of health inequalities reflect the characteristics of the underlying social welfare regime [27–29].

Socioeconomic uncertainties, such as the collapse of the Soviet Union, have repeatedly been found to detrimentally affect population health [30–32]. Lokshin and Ravallion [33] endorse the contextual relevance of this phenomenon. Between 1991 and 1992, real per capita income in Russia fell by 40 % to 1970s levels, and in 1993, the income gap between the highest and the lowest deciles of the population was elevenfold [34]. The macroeconomic changes of the period resulted in the impoverishment of much of the Russian population [35].

The relationship between poverty and the poor health status of the Russian Federation’s population has been clearly established [36]. A study by Bobak et al. [37] found that education and material deprivation are important predictors of self-assessed health with large socio-economic gradients. A drop in male life expectancy between 1990 and 1994, a radical unfavourable shift in mortality among adult working men, and a steep decline in fertility among women after 1992 are explained by the Robin Hood Index; these phenomena result from macroeconomic uncertainty and the widening income difference among Russia’s regions [38].

The self-assessed health of Russian adults shows very little gradient with household consumption or income [39, 40]. Lokshin and Ravallion [39] argue that, for Russians, a steeper gradient is emerging between subjective health predicted on the basis of specific objective health indicators and self-rated economic welfare. Furthermore, they suggest the existence of factors influencing self-rated economic welfare that are independent of current incomes and expenditures in the Russian Federation [41].

This study provides an insight into the interaction between ecological (macro) factors and individual (micro) factors in the Russia Federation from the 1990s onwards. We investigate the extent of health differences related to SES. Firstly, we examine the inequality in the distribution of health between worse-off and better-off Russians over the period 1994–2012. Secondly, we identify the association between the self-perceived health status of Russians and the variables related to SES between 1994 and 2012. Finally, we explain health inequality using the concentration index and subsequently, decompose the concentration index [42] to examine the contribution of factors related to SES on health differences. The concentration index reflects the experiences of the entire population and it is sensitive to the distribution of the population across socioeconomic groups. The regression-based decomposition of the annual concentration index into the contribution of age, gender, income, working status, living standard, geography of residence, asset-holding, and wealth possession allows us to distinguish whether the changes in health differences stem from the distribution of these variables, from changes in the association of these variables with self-perceived health, or from changes in the means of these variables [8]. So, in a single sweep, the decomposition provides a way not just of *explaining* inequality but also of *measuring* inequity.

## Methods

We used 18 waves of cross-sectional and panel data (where a portion of the households were followed over time) from the Russian Longitudinal Monitoring Survey (RLMS: 1994–2012). The Russian Longitudinal Monitoring Survey (RLMS: sourced with permission to use from Donna Miles, Senior Programmer, and Klara Peter, Associate Professor, Carolina Population Center, University of North Carolina) is a series of nationally representative surveys designed to monitor the effects of reforms on the health and economic welfare of households and individuals in the Russian Federation. The RLMS represents the first nationally representative random sample for Russia, albeit a highly clustered one – the mean cluster size in the entire sample is about 42. RLMS applies a multi-stage sampling method with pre-computed cross-sectional post-stratification weights. These weights adjust not only for design factors^2^ but also for deviations from the census characteristics.^3^ The overall response rate exceeded 70 % for households and 80 % for individuals within the participating households (http://www.cpc.unc.edu/projects/rlms-hse).^4^

The total number of observations in all 18 surveys was 220,052 (female: 57.25 % and male: 42.75 %). After excluding observations with missing information, we were left with 198,136 observations (Table 1. female: 57.27 % and male: 42.73 %). Welfare-related health inequality is best expressed with a concentration index (CI). The CI ranks members of the population according to their position in the underlying socioeconomic welfare distribution and correlates this welfare rank with individual health [43, 44].

**Table 1:** Sample size by year

Survey year	Total	*N* (Male & Female, %)	Observations with missing information (%)
1994	11,289	8,864 (43.87 & 56.13)	21.48
1995	10,664	8,359 (43.38 & 56.88)	21.61
1996	10,468	8,308 (43.12 & 56.88)	20.63
1998	10,677	8,664 (43.28 & 56.72)	18.85
2000	10,976	9,049 (42.99 & 57.01)	17.56
2001	12,128	10,076 (42.65 & 57.35)	16.92
2002	12,526	10,480 (42.89 & 57.11)	16.33
2003	12,659	10,606 (42.98 & 57.02)	16.22
2004	12,653	10,612 (42.97 & 57.03)	16.13
2005	12,238	10,310 (42.97 & 57.03)	15.75
2006	12,497	12,443 (42.78 & 57.22)	0.43
2007	12,302	12,245 (42.65 & 57.35)	0.46
2008	11,864	11,834 (42.10 & 57.90)	0.25
2009	11,816	11,777 (42.18 & 57.82)	0.33
2010	18,305	17,748 (42.53 & 57.47)	3.04
2011	18,302	18,203 (42.49 & 57.51)	0.54
2012	18,688	18,558 (42.08 & 57.92)	0.70
Total (*N*)	220,052	198,136 (42.73 & 57.27)	9.96
Annualized mean	12,944	11,655	9.96

A subjective measure of health was used in our analysis. Individuals were asked, “How would you evaluate your health?”, and the responses were captured on a five-point Likert scale with the following answers: “Very good”, “Good”, “Average – not good but not bad”, “Bad”, and “Very Bad”. Measuring the CI requires a cardinal health measure. We collapsed the five-scale measure into a binary variable [45–52], “self-perceived health” (1 = “Very good”, “Good”, and “Average – not good but not bad”; 0 = “Bad” and “Very Bad”). Age in years and gender are included as control variables in all analyses.

Conventionally, economists tend to favour a conception of welfare as a proxy for well-being in a broader sense, traditionally expressed by income and consumption. Both are important enabling factors for improving the non-material dimensions of living standards. However, the exclusion of the living standard measures in multivariate analysis raises the possibility that other coefficient estimates are biased [53]. Hence, the use of data on household assets and other characteristics have been used to construct alternative measures of welfare or living standards [54, 55]. In addition, asset and wealth data are likely to be less prone to fluctuation than consumption or income; therefore, they are considered a better reflection of long-term household wealth or welfare standards. In addition, at an empirical level, the correlation between consumption and the asset index is often weak [56].

Income [57], working status [28, 58, 59], geography of residence, access to publicly provided services, wealth possession, and asset-holding [53] were used as the variables of SES. Using these variables, we constructed a multidimensional indicator [60] to examine the role of different forms of deprivation in self-perceived health status for the individual. The income variable represented the sum of incomes from all sources for the household and it was deflated to the value of June 1992. We calibrated the household income as per adult equivalent using the OECD scale [AE = 1 + 0.7 (N_adults_ – 1) + 0.5 N_children_] for our analyses. The “geography of residence” variable distinguished the urban from the rural population. The “living standard” variable separates housing with and without amenities such as central heating, a hot running water supply, sewerage connectivity, and fixed-line telephone services. The “wealth possession” variable measures the net material worth of the household estimated by ownership of real estate property and savings. The “asset-holding” [54] variable measures the possession of durable goods (television, washing machine, car, and similar items).

We standardized self-perceived health status by age and gender, applying the indirect method of standardization [61, 62]. We estimated the correlation of the confounding variables (age and gender) with health conditional on non-confounding variables (education, working status, and geography of residence). This regression-based approach (Appendix 1) “corrects” the actual distribution of self-perceived health status by comparing it to the distribution that would be observed if all individuals in the group had their own age and gender characteristics but the same mean age and gender effect as the entire population. We compared the mean of self-perceived health status with that of standardized self-perceived health status by income quintiles (Table 4).

In the next step of the analysis, we used a random effect model (Appendix 2) to explain the perceived health of the respondents. The 18-year period of observations (1994–2012) in our datasets is sufficient reason to assume that the differences across individuals over the years exerted sufficient influence on self-perceived health status.

We used the health concentration index (Appendix 3) as our measure of SES-related health inequality. The concentration index ranks individuals by SES position rather than by health, and thus ensures that the socioeconomic dimension of inequalities in health is taken into account [42].

Finally, we decomposed (Appendix 4) the concentration index to estimate the contribution of different determinants in the production of health differences across SES.

## Results

Table 2 presents the characteristics of the survey population for 1994, 2000, 2006, and 2012. The distribution of respondents by age group remained almost same for all waves in the survey datasets. The proportion of female respondents increased consistently over time. Inflation-adjusted net equivalent household income increased by almost 80 % over the 18-year period, with an interim fall of almost 36 % in 2000 when compared to 1994. This trend corresponded with the reduced number of employed respondents. Further, in 2000, the proportion of urban respondents was also smaller than that of 1994, although the overall distribution of respondents between urban and rural areas did not change greatly over the period.

**Table 2:** Descriptive statistics of demography and socioeconomic characteristics for 2012, 2006, 2000, and 1994

	2012 (*N = 18,558*)		2006 (*N = 12,443*)		2000 (*N = 9,049*)		1994 (*N = 8,864*)	
Age group	Male (%)	Female (%)	Male (%)	Female (%)	Male (%)	Female (%)	Male (%)	Female (%)
<30	13.59	15.31	15.48	16.71	15.05	16.99	13.47	14.68
31–44	11.26	13.46	10.70	12.43	11.40	13.08	13.19	14.77
45–60	10.56	14.65	10.70	14.66	9.15	12.06	10.1	12.92
61–74	4.89	9.45	4.40	8.71	6.27	10.86	6.0	9.7
≥75	1.79	5.05	1.49	4.71	1.12	4.02	1.12	4.06
Total (%)	42.08	57.92	42.78	57.22	42.99	57.01	43.87	56.13
Household size (mean)	4.26**		4.35^		4.04^^		3.41*	
Working status (employed %)	56.16		54.25		49.52		54.66	
Adult equivalent household mean income (roubles) per month^a^								
Male (head)	7,690.00		5,290.00		2,730.00		4,280.00	
Female (head)	7,360.00		4,990.00		2,510.00		4,080.00	
Asset-holding (%)								
All durable assets^b^ but no car or tractor	88.23		65.19		77.74		68.19	
All durable assets with car and/or tractor	11.42		30.92		19.31		29.28	
Positive wealth group^c^ (%)	89.87		86.64		90.71		92.73	
Living standard (access to all publicly provided services) %	95.25		91.69		86.81		83.36	
Geography of residence (%)								
Urban	68.06		68.79		65.12		70.23	
Rural	31.94		31.21		34.88		29.77	

*std. deviation = 1.52; **std. deviation = 2.48; ^std. deviation = 2.49; ^^std. deviation = 2.09

^a^Individual income is a flawed metric of individual command over commodities, given that there is some degree of income pooling within households [38]

^b^Possession of a television, washing machine, and similar items; also known as white goods

^c^Respondents who own real estate with or without agricultural produce and with or without savings made in the last 30 days

Between 1994 and 2012, the proportion of respondents with all durable assets including a car and/or tractor decreased, while the proportion of respondents with all durable assets excluding a car and/or tractor increased. Respondents with no wealth consistently increased, but the number of respondents with access to all publicly provided services increased consistently during the study period (Table 2).

Table 3 presents the distribution of below average (bad and very bad) self-perceived health status by age group and gender, geography of residence, and income quintile. Overall, self-perceived health status for both genders across all age groups improved over the study period. However, male respondents under 60 years of age with a below-average self-perceived health status were greater in number in 2000 when compared to 1994. The proportion of respondents with average and above-average self-perceived health increased by almost 36 % over the 18-year period. On the one hand, the respondents with a diagnosed chronic disease had a worse self-perceived health status in 2012 compared to 1994, but on the other hand, a relatively higher proportion of female respondents with average and above-average self-perceived health had a diagnosed chronic disease.

**Table 3:** Descriptive statistics of self-perceived health status for 2012, 2006, 2000 and 1994

Below average (bad and very bad)self-perceived health by age group	2012 (*N = 18,558*)		2006 (*N = 12,443*)		2000 (*N = 9,049*)		1994 (*N = 8,864*)	
	Male (%)	Female (%)	Male (%)	Female (%)	Male (%)	Female (%)	Male (%)	Female (%)
<30	1.70	2.01	2.39	2.84	2.79	4.36	2.60	5.53
31–44	3.40	3.80	3.98	6.59	6.49	9.12	5.82	10.77
45–60	10.06	13.46	11.79	15.41	15.34	18.42	14.53	26.90
61–74	24.45	30.69	29.74	38.75	34.22	45.98	37.41	49.30
≥75	42.17	56.08	54.05	65.36	60.40	62.64	60.61	68.06
Total (%)	8.62	14.72	9.75	17.49	12.52	20.47	12.55	23.92
Total below average (bad and very bad) self-perceived health (%)	12.15		14.18		17.05		18.93	
Respondents (both genders) diagnosed chronic disease by self-perceived health (%)								
Very good	0.84	4.44	2.88	12.35	0.70	1.28	0.81	2.38
Good	0.83	3.20	0.65	4.67	1.22	4.17	1.22	3.32
Average	7.38	13.97	6.27	11.84	5.74	11.60	5.85	10.73
Bad	33.04	41.30	33.86	34.87	27.32	26.74	26.03	25.75
Very bad	58.95	59.39	45.21	44.72	35.06	46.86	38.96	40.18
Total	6.97	14.95	6.91	14.44	6.89	13.76	7.02	13.63
Total respondents with diagnosed chronic disease (%)	11.59		11.22		10.81		10.73	
Below average (bad and very bad)self-perceived health by geography of residence (%)								
Urban	8.85	14.17	9.75	16.56	12.82	19.47	12.11	22.82
Rural	8.13	16.78	13.20	19.53	11.96	22.35	13.49	26.60
Total (%)								
Urban	11.94		16.56		16.63		18.15	
Rural	12.92		19.53		17.84		20.78	
Below average (bad and very bad)self-perceived health by income quintile (%)								
Poorest	9.98		14.61		19.28		22.34	
Second-poorest	12.89		19		18.77		22.59	
Middle	14.58		14.79		11.78		18.69	
Second-richest	15.08		12.75		12.45		15.11	
Richest	9.28		9.92		10.13		9.85	
χ^2^ (chi-squared)	0.000		0.000		0.000		0.000	

The changes in self-perceived health status for both urban and rural respondents registered a similar trend from 1994 to 2012. The difference in self-perceived health status between urban and rural respondents reduced substantially between 1994 and 2012. In the middle and lower income quintile, the proportion of respondents with below-average self-perceived health status decreased substantially in 2012 when compared to 1994 (Table 3). Surprisingly, the self-perceived health status of the two poorest quintiles had improved most over the 18-year period.

The difference between the age- and gender-standardized mean self-perceived health status and mean self-perceived health status reduced from 1994 to 2012 when the effect of education, working status, and the geography of residences were controlled for (Table 4). For the three higher income quintiles, there was an upward shift in the difference in 2012 after a decline in difference from 1994 to 2006. The differences in age- and gender-standardized means between the income quintiles were smaller in 2012 than in 1994.

**Table 4:** Age- and gender-standardized self-perceived health status (1 = average and above-average self-perceived health and 0 = bad and very bad self-perceived health)

Income quintile	2012 (*N = 18,558*)			2006 (*N = 12,443*)			2000 (*N = 9,049*)			1994 (*N = 8,864*)		
	std. mean*	mean	∆**	std. mean*	mean	∆**	std. mean*	mean	∆**	std. mean*	mean	∆**
Poorest	0.912	0.900	0.012	0.875	0.854	0.021	0.871	0.807	0.064	0.854	0.777	0.077
Second-poorest	0.895	0.871	0.024	0.841	0.810	0.031	0.884	0.812	0.072	0.865	0.774	0.091
Middle	0.890	0.854	0.035	0.880	0.852	0.028	0.935	0.882	0.053	0.896	0.813	0.082
Second-richest	0.892	0.849	0.043	0.895	0.872	0.023	0.921	0.876	0.046	0.920	0.849	0.071
Richest	0.942	0.907	0.035	0.920	0.901	0.019	0.936	0.899	0.037	0.956	0.902	0.054
Total	0.912	0.877	0.035	0.882	0.857	0.025	0.891	0.829	0.062	0.888	0.810	0.079

*std. mean = indirectly standardized mean self-perceived health status

**the difference between the age- and gender-standardized mean self-perceived health status and the mean self-perceived health status

Female respondents consistently reported relatively worse health than men did during the study period (Table 5). Table 5 shows that unemployment was, *ceteris paribus*, associated with a more than 8 % higher risk for having bad health when compared to being employed. The smoking habits of the individual, household size, adult equivalent household income, wealth possession, and living standard had significant associations with self-perceived health status. Having a chronic disease had a significant negative association with self-perceived health status in the model, as one might expect. From the 2000 onwards, self-perceived health status improved consistently over time (Table 5).

**Table 5:** Panel data logistic model for self-perceived health status (1 = average and above-average self-perceived health and 0 = bad and very bad self-perceived health), random effects

Variables	Basic model (constant only)	Model with individual attributes	Model incl. SES variables
Gender (Female = 1)		−0.028***	−0.024***
Age		−0.007***	−0.007***
Chronic diseases (1 = yes; 0 = no)		−0.167***	−0.166***
Smoking (1 = yes; 0 = no, incl. former smokers)		0.004***	0.000***
Household size		0.003***	0.002***
Adult equivalent household income (roubles)			0.000***
Work status (1 = working; 0 = not working)			0.082***
Wealth group (1 = possession of wealth; 0 = no wealth)			0.007**
Living standard (1 = access to all publicly provided services; 0 = no access to publicly provided services)			0.012**
Asset-holding (comparison group = no durable assets)			
Durable assets without car/tractor			0.056***
Durable assets with car/tractor			0.012
Comparison year: 1994			
1995	0.008	0.008*	0.009*
1996	0.000	0.003	0.006
1998	−0.012*	0.002	0.006
2000	−0.021***	0.007	0.009*
2001	−0.017***	0.018***	0.019***
2002	−0.020***	0.020***	0.020***
2003	−0.030***	0.016***	0.015***
2004	−0.030***	0.023***	0.021***
2005	−0.030***	0.028***	0.024***
2006	−0.035***	0.030***	0.034***
2007	−0.033***	0.034***	0.034***
2008	−0.046***	0.027***	0.022***
2009	−0.048***	0.031***	0.026***
2010	−0.038***	0.046***	0.039***
2011	−0.045***	0.045***	0.037***
2012	−0.035***	0.059***	0.049***
Intercept	0.887***	1.175***	1.051***
N	198,136	197,951	187,540
R^2^			
-within	0.009	0.02	0.024
-between	0.014	0.31	0.352
-overall	0.002	0.22	0.259
ρ	0.486	0.337	0.300
Prob > χ^2^	0.000	0.000	0.000

*p < 0.05, **p < 0.01, ***p < 0.001

Of the SES-related variables, durable asset-holding had a significant association (Table 5) with self-perceived health status (precisely one additional unit of durable asset-holding increases positive self-perceived health status by more than 5 % when all other variables are kept constant). However, the possession of goods such as a car or tractor did not have a significant association. In the panel data model (Table 5), the intra-class correlation (ρ) was 0.30. A small value of ρ implies that although there was a statistically significant difference in self-perceived health status between individuals, there was also large variation in the self-perceived health status of the individual respondents over the long follow-up time of our study.

A positive concentration index indicated a concentration of average and above-average self-perceived health among better-off respondents (Table 6). Table 6 presents the factor contribution [$\left({\beta}_K{\overline{X}}_K/\mu \right)\ {C}_k$] to SES-related health inequalities for 1994, 2000, 2006, and 2012. A negative contribution of a factor to the concentration index indicates [Appendix 4: Eqn. 8] that the factor correlates positively with self-perceived health status, and such a contribution is concentrated among individuals with lower SES status (more material deprivation); likewise, the reverse is true. Thus, bad and very bad self-perceived health accumulates among the worse-off [8]. The value of the concentration index for self-perceived health status increased from 1994, but it was found to be stable in subsequent years.

**Table 6:** Health inequity indices and decomposition

	Total change (1994–2012)		2012		2006		2000		1994	
	contrib. (%)		contrib. (%)		contrib. (%)		contrib. (%)		contrib. (%)	
Gender (female)	−1.147	0.187	0.05	0.018	0.00	0.000	1.739	−0.055	1.19	−0.169
Age	−6.660	−0.164	9.84	−1.319	17.67	−0.878	4.518	−1.236	16.50	−1.155
Working status	43.071	0.342	53.08	0.524	14.23	0.197	27.325	0.315	10.01	0.182
Income	−14.999	−0.204	17.83	0.526	−13.02	−0.272	65.521	1.292	32.83	0.729
Geography of residence	−9.349	−0.167	15.33	0.232	72.61	0.926	4.565	0.112	24.68	0.399
Living standard	−5.869	−0.284	−4.25	−0.250	13.37	0.547	−2.823	−0.093	1.62	0.034
Wealth possession	−0.250	−0.040	−0.14	−0.017	−0.82	0.158	−0.764	−0.064	0.11	0.022
Asset-holding	−4.986	0.069	8.06	−0.254	−4.03	0.090	−0.080	0.003	13.05	−0.323
Concentration index (standard error)	0.001		0.008 (0.002)		0.008 (0.002)		0.008 (0.003)		0.007 (0.003)	
Gini index	−0.103		0.366		0.392		0.451		0.469	

= elasticity; contrib. = contribution

The negative contribution of living standard and wealth possession in 2000 and 2012 implied that the concentration of living standard (access to all publicly provided services) and wealth possession among the better-off had increased the concentration of bad and very bad self-perceived health amongst the worse-off. Similarly, a higher income and asset-holding in 2006 were associated with lower risks of bad and very bad self-perceived health, and these factors were concentrated among the better-off. The positive contribution of age in all the years moderated the observed inequality; elderly individuals were vulnerable to a higher risk even if they were members of the better-off SES. Gender did not contribute significantly to the health gradient.

In the decomposition of the total change in the concentration index between 1994 and 2012, working status, income, geography of residence, and living standard were the most important variables in their contribution to SES-related health inequalities. The relative contribution of working status increased fivefold, while the relative contribution of income reduced by almost a half over the 18-year period. Again, the change in the elasticity effect of working status, income, geography of residence, and living standard on the contribution to the concentration index was also evident from 1994 to 2012. The change in the Gini index indicated an improvement in the distribution of SES-related variables from 1994 to 2012.

**Fig. 1:**
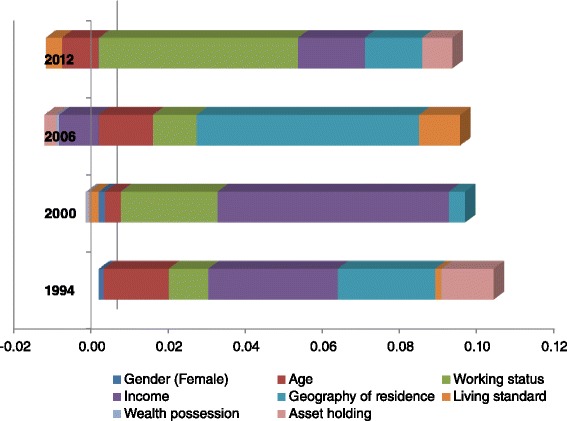
Decomposition of changes in health inequalities

Figure 1 illustrates the factor-level effect (marginal effects of explanatory variables evaluated at sample means) of the four most important SES-related factors on health concentration index for 1994, 2000, 2006 and 2012. The factor-level [$\left({\beta}_K{\overline{X}}_K/\mu \right)\ {C}_k$] effect reflects the change of the concentration index (health inequalities) of self-perceived health as bad and very bad that was numerically induced by the change of the variable’s (SES-related) mean. In 2000, income was the dominant variable, in 2006, the geography of residence was dominant, and in 2012, working status was dominant. The negative factor level effect of living standard and wealth possession in 2000; income, wealth possession, and asset-holding in 2006; and age and living standard in 2012 indicated that a reduced effect (direct effect of *β*_*K*_ on *C*_*k*_ and the indirect effect operating through *μ*) of the specific determinants of health had decreased the degree of inequality in self-perceived health for the respective year.

## Discussion

This paper addressed inequalities related to socioeconomic strata (SES) in self-perceived health over a period of 18 years (1994–2012) in the Russian Federation. Over this time, the perceived health status of Russians improved. There was a systematic trend of improvement in the age- and gender-standardized mean of self-perceived health status after controlling for the effect of education, working status, and the geography of residence among the worse-off. Bobak et al. [37] found that the distribution of observed self-perceived health status of the Russian population was in the same range as other former-Soviet countries.

On examining the inequality in the distribution of health between worse-off and better-off Russians over the period, we found that the difference in standardized mean self-perceived health status between the richest and the poorest income quintiles reduced from 0.10 to 0.03. This 30 % reduction in the health gap could be attributed to a more even distribution of variables related to SES in 2012 (measured as Gini index values).

The attempt to identify the association between the self-perceived health status of Russians and the variables related to SES between 1994 and 2012 revealed inter-individual health differences by level of interaction between ecological (macro) and individual (micro) factors. The individual variances in self-perceived health status were found to be higher than the variances between individuals over the period. The association between self-perceived health status and the possession of durable assets (other than a car and/or tractor) were found to have the strongest association among other variables related to SES (household size, income, working status, wealth possession, and living standard).

A positive change in the standardized mean of self-perceived health status from 0.88 in 1994 to 0.91 in 2012 could be due to the effect of employment, income, asset-holding, wealth possession, and living standard (access to all publicly provided services). Consistently from 2001 onwards, there were also statistically significant changes in the association of self-perceived health status with household size, income, working status, wealth possession, living standard, and asset-holding. In line with an earlier study [39], we found that adults living in larger households tend to have better self-perceived health status.

Finally, we measured the health inequality index (concentration index), and subsequently decomposed the concentration index [42] to examine the contribution of factors related to SES on health differences. A positive change in health inequality index (from 0.007 in 1994 to 0.008 from 2000 onwards), indicated a change towards better health for the better-off – a finding consistent with the results of Lokshin and Ravallion [39]. The negative change in the Gini index (over 10 %) reflected a better distribution of variables related to SES in 2012 compared to 1994. This improved distribution follows Ivanter’s findings [63], showing the continuing process of the restoration of income to pre-1998 levels (1998 was the year of the second economic crisis after the disintegration of the USSR) – the mass income group showed stabilization from 2006 onwards [63].

The decomposition results of the concentration index suggested the following as the most important contributors to health differences: working status in 2012, geography of residence in 2006, and income in 2000 and 1994. The high contribution of working status (being employed) to perceived health status reflected the increasing distribution effect of working status. The association of no work with a higher risk for poor health was consistent with previous studies [6, 28, 58, 59]. Gavrilova et al. [64] and Brainerd and Cutler [65] also argue that the “psychosocial stresses” of the transition in Russia are a more important cause of the health crisis than poverty. However, this stress may well make it hard to identify the importance of poverty. The effect of income on perceived health was concentrated among the better-off. This income and health relationship with its distribution supports Ivanter [63], who argues that the income-level improvement after the crisis in 1998 is continuing in general, but money tends to concentrate among the higher income groups. The effect of income was also evident in 1994, albeit to a relatively lesser extent (the first economic crisis after the disintegration of the USSR occurred in 1992).

The strong association of geography of residence (urban and rural) with perceived health status in 2006 demonstrated the concentration of health among the better-off. The age effect of the concentration index placed elderly individuals in a higher SES, and this distribution effect reinforced the findings of Ivanter [63], who states, “the majority of qualified workers are, unfortunately, above 55 or sometimes 65, and there are no replacements for them”. Bad and very bad self-perceived health status decreased among the urban population and female respondents of the worse-off part of the population in 2012 when compared to 1994.

This study has a number of strengths. First, it uses the most recent datasets available. Second, it is not restricted to the cross-sectional approach of a one-year survey; instead, it investigates the evolution of health over a long period of time using 18 waves of cross-sectional and panel data (some households were followed over time). Third, the study decomposes the total observed health differences into the contribution of health elasticity and inequality by SES-related health determinants.

The study also has some limitations. First, the use of survey data usually involves a potential for biases owing to non-response: our average rate of excluded observations is presented in Table 1. Second, cross-sectional data have the potential for reverse causation between the variables of SES and health, and the results may reflect the reverse effects of health on SES. Third, there is the potential for bias that is intrinsic to subjective data: responses to the questionnaire on self-perceived health are often correlated with variables of SES and other observables [66].

## Conclusions

We investigated the association of variables related to socioeconomic strata (SES) in health differences among Russians. There is an evident gender difference in the socioeconomic covariates of health. Health is often studied as a binary variable (such as average and above-average self-perceived health vs bad and very bad self-perceived health) in the literature [45–52]. We consider our results fairly robust, since we find the presence of chronic disease risks among respondents with bad and very bad self-perceived health. Our results are also consistent with the views that the health gap between the worse-off and better-off is underestimated, as factors that influence welfare are ignored. To conclude, self-perceived health differences related to SES have changed in the Russian Federation over time; this can largely be attributed to changes in the contributions of individual characteristics that represent labour market position, income, access to all publicly provided services, geography of residence, possession of durable assets, and household size. Further, such changes in self-perceived health status stems from both changes in the distribution of the determinants of health as well as from changes in their association (effects operating through the mean) with self-perceived health status. Thus the decomposition analysis has provided the measurements of inequality. Hence, beyond explaining inequalities, our study guides the policy intervention for choosing the determinants in addressing the problem of health inequalities for the Russian population. Overall, this study supports Coburn [67] who argued that health inequalities are largely determined by socioeconomic and political contexts.

## Appendix 1

1 $$ {y}_i=\alpha +{\displaystyle {\sum}_j}{\beta}_j{\mathrm{x}}_{ji}+{\displaystyle {\sum}_k}{\gamma}_k{z}_{ki}+{\epsilon}_{i,} $$

where, *y*_*i*_ is self-perceived health status; *i* denotes the individual; and α, β, and γ are parameter vectors. The x_*j*_ are confounding variables (age and gender) that we want to standardize, and the *z*_*k*_ are nonconfounding variables (education, working status and geography of residences) that we do not want to standardize but to control for in order to estimate partial correlations with the confounding variables. The Newey–West estimator 5 estimates $\left(\widehat{\alpha},{\widehat{\beta}}_j,\ {\widehat{\gamma}}_k\right)$ the individual values of the confounding variables (x_*ji*_), and sample means of the non-confounding variables $\left({\overline{z}}_k\right)$ are then used to obtain the predicted, or “x-expected,” values of the self-perceived health status *ŷ*_*i*_^*x*^.

2 $$ {\widehat{y}}_i^x=\widehat{\alpha}+{\displaystyle {\sum}_j{\widehat{\beta}}_j{\mathrm{x}}_{ji}}+{\displaystyle {\sum}_k}{\widehat{\gamma}}_k{\overline{z}}_k. $$

Estimates of indirectly standardized self-perceived health:

3 $$ {\widehat{Y}}_i^{IS}={Y}_i - {\widehat{Y}}_i^X + \overline{Y} $$

where,

- *Ŷ*_*i*_^*IS*^ = *indirectly standardized*, *self-perceived health status*
- *Y*_*i*_ = *actual health*
- *Ŷ*_*i*_^*X*^ = *x-expected health*
- $\overline{Y}=$ *overall sample mean*.

## Appendix 2

The model:

4 $$ {y}_{it}=\beta {\mathrm{x}}_{it} + \alpha + {\mu}_{it}+{\varepsilon}_{it} $$

where,

- *y*_*it =*_ *self-perceived health status (dependent variable); i = individual; t = time*
- x_*it*_ *= predictor variable*
- *β* = *coefficient for the predictor variable*
- *α* = *unknown intercept for each individual*
- *μ*_*it*_ = *between-individual error*
- *ε*_*it*_ = *within-individual error*.

The model use was tested for model selection (fixed vs random effects) with the Hausman test, and with the Breusch and Pagan Lagrangian multiplier (LM) test for random effects regression. The model use was also tested for misspecification with the RESET test; it does not suffer from any problems with misspecification or omitted variables.

We included multidimensional indicators of the variables of SES in the model. We tested the potential multicollinearity (variance inflation factor; all values were less than 5), and thus the chosen variables of SES did not correlate with each other. Furthermore, we tested for autocorrelation (the Wooldridge test, no first-order autocorrelation was observed). The Breusch-Pagan/Cook-Weisberg test found no heteroscedasticity in the data.

## Appendix 3

The concentration curve plots the cumulative proportion of self-perceived health (y) against the cumulative share of the population ranked by SES variables. The curve lies below the 45° line (diagonal) of equality if health is concentrated among the better-off, and above the 45° line (diagonal) of equality if health is concentrated among the worse-off. The concentration index is defined as twice the area between the concentration curve and the diagonal, i.e. $C=\frac{2}{N\mu }{\displaystyle {\sum}_{i=1}^N}{w}_i{y}_i{R}_i-1$; C is bounded between −1 and +1 and μ is the weighted mean self-perceived health of the population (*N*). *N* is the sample size, *w*_*i*_ is the sampling weight of individual *i* (with the sum of *w*_*i*_ equal to *N*), and *R*_*i*_ is the fractional rank of the *i* individual.

5 $$ \mu = \frac{1}{N}{\displaystyle {\sum}_{i=1}^N}{w}_i{y}_i $$

6 $$ {R}_i={\displaystyle {\sum}_{j=1}^{i=1}}{w}_j+\frac{1}{2}{w}_i $$

where *w*_0_ = 0

*R*_*i*_ denotes the weighted cumulative proportion of the population up to the midpoint of each individual weight and is bounded in the (0;1) interval. *R*_*i*_ represents the cumulative distribution function of income and indicates the individual’s position within the income distribution. C becomes positive if health is concentrated among the better-off, negative if health is concentrated among the worse-off, and zero if no inequality is observed. Thus, C is computed [42] with the weighted covariance of *μ* and the fractional rank *R*_*i*_ as

7 $$ \mathrm{C}=\frac{2}{N\mu }{\displaystyle {\sum}_{i=1}^N}{w}_i\left({y}_i-\mu \right)\left({R}_i-\frac{1}{2}\right) = \frac{2}{\mu }co{v}_w\left({y}_i{R}_i\right). $$

## Appendix 4

Based on the assumption of a linear additive relationship between the health variable y and a set of explanatory variables x, i.e. y_*i*_ = *α* + ∑_*k*_ *β*_*k*_ *x*_*ki*_ + *ε*_*i*_ [*x*_*k*_ are sets of health determinants and *ε* is the disturbance term], we used the framework [8] to decompose the concentration index for y when C is expressed as

8 $$ \mathrm{C}={\displaystyle {\sum}_{\mathrm{k}}}\left({\upbeta}_{\mathrm{K}}{\overline{\mathrm{X}}}_{\mathrm{K}}/\upmu \right){\mathrm{C}}_{\mathrm{k}} + {\mathrm{GC}}_{\upvarepsilon}/\upmu $$

where,

$$ {\overline{X}}_K $$ *= mean of x*_*k*_

*C*_*k*_ *= concentration index for x*_*k*_ *(defined analogously to C)*

*GC*_*ε*_ *= generalized concentration index for the disturbance term.*

Thus, concentration index C is equal to a weighted sum of the k regressors. The weight for regressor k is the elasticity of y for *x*_*k*_. The residual component reflects health inequality not explained by systematic variation across income groups in the regressors.

The estimated health elasticity of determinant k is written as ${\widehat{\eta}}_k=\left({\widehat{\beta}}_K{\overline{X}}_K/\mu \right){C}_k$, ${\widehat{\eta}}_k$ = the relative change of y statistically associated with a one-unit change of the corresponding *x*_*k*_.^6^ Hence, the weighted sum of inequality in each of the health determinants (with the weights equal to the health elasticities of the determinants) is the health inequality. Therefore, $\widehat{C}={\displaystyle {\sum}_k}{\widehat{\eta}}_k{C}_k$. Wagstaff et al. [8] argue that changing contributions can be caused either by changes in the elasticities of *η*_*k*_ or by changes in the distribution of *C*_*k*_ of *x*_*k*_.

## Footnotes

1 The readers are assumed to be familiar with the general economic and political ramifications of the transformation on transition [Svejnar, J. Transition economies: performance and challenges. J. Econ. Perspect.;2002; 16:3–28. Roland, G. Polit. Econ. Transit. J. Econ. Perspect.; 2002; 16:29–50. Boeri, T. and Terrell, K.. Institutional determinants of labor reallocation in transition. J. Econ. Perspect.; 2002; 16:51–76. Berglof, E. and Bolton, P. The great divide and beyond: financial architecture in transition. J. Econ. Perspect.; 2002; 16:77–100. Estrin, S. Competition and corporate governance in transition. J. Econ. Perspect.; 2002; 16:101–124].

2 Correct non-coverage biases in the frame used to derive the original sample of dwellings and individuals.

3 The RLMS sample compared favourably to those determined by the Soviet census: there is usually a difference of only one percentage point or less between the two distributions.

4 Because of the decline in response rate in big cities, the proportion of the big cities in the sample became less than required and continued to decrease with each round, so in Round 15 a sample repair was performed.
